# Beyond clinical scales: an observational study on instrumental gait analysis and biomechanical patterns in patients with Parkinson’s disease

**DOI:** 10.3389/fbioe.2025.1541240

**Published:** 2025-04-30

**Authors:** Paolo De Pasquale, Mirjam Bonanno, Cristiano De Marchis, Luca Pergolizzi, Antonino Lombardo Facciale, Giuseppe Paladina, Maria Grazia Maggio, Federica Impellizzeri, Irene Ciancarelli, Angelo Quartarone, Rocco Salvatore Calabrò

**Affiliations:** ^1^ IRCCS Centro Neurolesi Bonino-Pulejo, Messina, Italy; ^2^ Department of Engineering, University of Messina, Messina, Italy; ^3^ Department of Life, Health and Environmental Sciences, University of L'Aquila, L’Aquila, Italy; ^4^ Department of Biomedical, Dental Sciences and Morphological and Functional Images, University of Messina, Messina, Italy

**Keywords:** gait analysis, biomechanics of gait, optoelectronic motion capture system, Parkinson’s disease, fear of falling, neurorehabilitation gait analysis, neurorehabilitation

## Abstract

**Introduction:**

Parkinson’s disease (PD), a common neurodegenerative disorder affecting motor functions, is associated with abnormal gait patterns characterized by altered kinematic, kinetic, and electrophysiological parameters. This observational study aims to instrumentally identify and quantify these gait dysfunctions in PD patients compared to normal values from healthy subjects.

**Methods:**

Sixty-nine PD patients underwent clinical and instrumental evaluations to assess gait. Demographic and clinical data were collected before motor assessment. Clinical scales evaluated the level of impairment, gait, balance, risk of falls and ability to complete activities of daily living. Instrumental evaluations were conducted using optoelectronic, force plates and electromyographic (EMG) systems in a motion analysis laboratory. Statistical analysis involved a non-parametric test to compare pathological and normal data, clustering methods to identify groups based on clinical evaluations, and a combination of non-parametric analysis and linear models to assess dependencies on clinical scales.

**Results:**

The results showed that PD patients had significant gait kinematic differences compared to normal values, with increased temporal and shortened spatial parameters. In addition, PD patients were grouped into four clusters based on clinical scales. While some gait features were influenced by clinical scales reflecting impairment, gait and balance, and independence, others were more affected by the perceived fear of falling (FoF).

**Discussion:**

In conclusion, the study identified specific biomechanical gait dysfunctions in kinematic, kinetic, and electrophysiological parameters in PD patients, undetectable by standard clinical scales. Additionally, higher FoF was associated with dysfunctional biomechanical patterns, independent of impairment severity, gait and balance dysfunction, or overall independence.

## 1 Introduction

Parkinson’s disease (PD) is one of the most common neurodegenerative disorders that affects the substantia nigra of the midbrain. PD is associated with motor, cognitive, and autonomic alterations (Hallett, 2014; Jellinger, 2014; Giustiniani et al., 2022). From the early stages of PD, patients may present dysfunctional gait patterns, mainly characterized by a shortened stride length, increased stride variability, reduced walking speed and festinating gait (Bonanno et al., 2023). The most detectable gait modifications in PD encompass various alterations, including decreased walking speed and step length, asymmetrical gait patterns, reduced arm swing, compromised dissociation between arm and trunk movements during ambulation, and postural instability (Zanardi et al., 2021). Pistacchi et al. (Pistacchi et al., 2017) found that reduced ankle flexion during the swing phase as well as in the stance phase, and mild knee flexion reduction can be detected even in early-stage PD patients. Some gait alterations in these patients are specific and get worse throughout the disease (Zanardi et al., 2021). Gait impairment in PD evolves as the disease progresses, showing distinct patterns at early, mild-to-moderate, and advanced stages. In the early phase, specific changes include reduced arm swing, less fluid movement, and increased asymmetry between limbs. Impaired muscle contraction, rigidity, and postural instability also contribute to reduced limb propulsion, negatively impacting gait parameters like speed and step length. Notably, reduced step length is a characteristic feature of Parkinsonian gait. As PD reaches the mild-to-moderate stage, symptoms become more symmetric, reducing earlier asymmetries. Gait difficulties intensify, with patients often exhibiting shuffling steps, prolonged double-limb support, and increased cadence. At this stage, motor automaticity further reduces, leading to fragmented movements, such as turning with a “block-like” motion (turning in block) and challenges with initiating steps. This pattern reflects both a loss of automaticity and impaired forward propulsion, hallmark characteristics of Parkinsonian gait. Together, these results further confirm that PD gait disturbances are characterized by slower, shorter, and less coordinated movements, with changes in both timing and spatial dimensions of movement that contribute to decreased mobility and stability. This reduced stability is consequently perceived in patients with PD as an enhanced fear of falls (FoF). According to Bryant et al., people with PD with higher levels of FoF tend to exhibit specific biomechanical gait features, such as slow gait speed, short stride length and balance deficits may predispose to falls (Bryant et al., 2014). In particular, gait speed is an important factor that should be considered in the rehabilitation training of Parkinsonian patients. Indeed, some authors (Paker et al., 2015) found that gait speed is strongly correlated with not only demographic factors (e.g., age, gender, height) but also with FoF, balance and mobility. In this sense, early identification and quantification of biomechanical gait parameters are key factors in establishing an effective and customized rehabilitation therapy (Biase et al., 2020; Zanardi et al., 2021). The quantification of disease progression in PD is mainly measured through the Hoehn and Yahr scale (H&Y) (Zhao et al., 2010) which is one of the most widely applied indices of disease severity. However, this clinical assessment tool is focused on gross motor functions, analysing roughly the patients’ quality of movements.

On the other hand, clinical scales or tests to assess gait functions are also often used in clinical practice to evaluate patients’ motor abilities, and “how” they walk, considering also the activities of daily living, and the level of fatigue (Biase et al., 2020). Specifically, this kind of assessment is commonly administered by physiotherapists to investigate the level of functional status of patients, before and after the rehabilitation path (Bonanno et al., 2023). Among the most used rating scales, in the PD population, are the Tinetti scale (TS) for gait and balance (Tinetti, 1986), and Berg Balance Scale (BBS) to assess static and dynamic balance (Berg et al., 1995), and the Falls Efficacy Scale-International (FES-I) to assess the perceived fear of falling (Caronni et al., 2022). However, clinical scales do not always reflect the “objective” stage of the disease, and they cannot detect specific movement changes that are not visible to the clinician’s eye, as some instrumental assessments do. Current approaches to evaluating objectively and quantitatively gait impairments include marker-based motion capture (MoCap) systems (Romero-Flores et al., 2024). In particular, optoelectronic tools are considered as the “gold standard”. These can use either passive systems consisting in a set of multiple cameras to track 3D trajectories (i.e., Vicon, Oxford UK), or active systems that can identify automatic movements through infrared light-emitting diode (LED) (i.e., Optotrack, NDI, Waterloo, ON, Canada), placed on different body landmarks. In this way, these systems allow a comprehensive recording of kinematics and kinetics. Additionally, they can be equipped with force platforms to detect ground reaction forces as well as electromyographic (EMG) sensors to record muscle activity. In this way, the conjunct use of both instrumented gait analysis (GA) and clinical scales became fundamental in the rehabilitation context, especially in PD patients, who manifest gait alterations at the early stages of the disease, but also to monitor the progression of the disease. Indeed, clinical scales, while valuable for assessing general symptoms and overall disease severity in PD, usually focus on broad aspects of motor symptoms (e.g., tremor, rigidity, bradykinesia) rather than specific gait parameters like spatial-temporal parameters and joint angles. These specific parameters are essential to understand the true nature of gait impairment in PD, but they are not well represented in most standard scales. In addition, gait alterations, such as slight reductions in step length or small increases in gait variability like in early stages of the disease, may go unnoticed in a clinical setting, where scoring often lacks the granularity needed to capture these early signs of impairment. For these reasons clinical scales cannot be the only assessment tool for PD motor evaluation. Another aspect that should not be neglected is that instrumental GA parameters could be more effective in detecting changes in biomechanical patterns of gait in PD patients after a rehabilitation program. In this context, Peppe and colleagues highlighted the efficacy of a rehabilitation intervention, which aimed at improving temporal parameters of gait, measured with instrumental GA (Peppe et al., 2007). These results could be valuable in creating a more objective assessment of motor rehabilitation programs and in demonstrating their impact on neurodegenerative conditions like PD. The findings on kinematic data of the lower limbs align with those documented in existing literature. In this sense, a complete assessment of gait, which comprises both instrumental and clinical measures, can be useful to personalize rehabilitation treatment according to patients’ needs. In addition, this combined approach can be also useful to monitor patients over time, during the progression of neurodegenerative disease.

In this observational study, our primary aim is to identify and quantify kinematic, kinetic, and EMG gait parameters obtained by a GA laboratory, which integrates optoelectronic MoCap system, force platforms and EMG sensors, in a group of PD patients, who are naive to gait training, compared to normal data.

Then, our secondary aim is to investigate biomechanical differences among PD patients by clustering them based on clinical rating scales (BBS, TS, Barthel index, H&Y and FES-I) and analysing variations in instrumental biomechanical data across these clusters.

## 2 Materials and methods

### 2.1 Study design and population

Sixty-nine PD patients, with a mean age of 66 ± 9 (see Table 1), were consecutively recruited from the Outpatient Movement Disorders Clinic of the IRCCS Centro Neurolesi “Bonino-Pulejo” (Messina, Italy) between June 2018 and March 2024.

**TABLE 1:** Socio-demographic and clinical data of the study sample.

N Subjects	69
Gender	
N Male (%)	50 (72)
N Female (%)	19 (28)
Age	66 ± 9
YSD	6 ± 4
BBS	41 ± 8
TS	20 ± 5
BI	77 ± 13
H&Y	3 ± 1
FES-I	33 ± 13

Legend: YSD, years since disease; BBS, Berg Balance Scale; TS, Tinetti Scale; BI, Barthel Index; H&Y, Hoen & Yahr; FES-I, Falls Efficacy Scale–International.

Patients were included if they: (1) had a diagnosis of PD according to the Movement Disorder Society Clinical Diagnostic Criteria for Parkinson’s Disease; (2) were naive to gait training; (3) were in “on” phase at the time of the evaluation and (4) were able to walk independently. Patients were excluded if they had: (1) cognitive, visual, or auditory deficits that could impair the comprehension and/or execution of the proposed motor tasks for the evaluation; (2) presence of comorbidities that affected upright posture and walking (e.g., hypotension); (3) refused consent or were unable to provide informed consent.

All experiments were conducted according to the ethical policies and procedures approved by the local ethics committee (IRCCS-ME-23/2022). All participants gave their written informed consent.

### 2.2 Procedures

All patients were assessed during their “on” phase while taking their own treatment dose, as per clinical indications. Evaluations took place in the morning, approximately 2 hours after the medication intake. Both the instrumented GA and clinical motor assessments were conducted on the same day as the routine neurological consultation by a multiprofessional team composed of a neurologist, a physiotherapist, a bioengineer and a motor science technician. This latter prepared PD patients for instrumented GA, the physiotherapist administered the clinical scales/tests, while the neurologist carried out the neurological examination. The GA and the subsequent analyses were performed by the bioengineer.

### 2.3 Clinical assessment

A skilled physiotherapist evaluated motor performances of PD patients using the following clinical scales/tests: i) BBS assesses static and dynamic balance using 14 tasks. The score ranges from 0 to 56, with scores below 40 indicating a moderate to high risk of falling; ii) TS is a 16-item measure (7 items for gait and nine for balance), where a total score of 18 or lower indicates a high risk of falls, and a score between 19 and 24 signifies a moderate risk of falls; iii) FES-I is a 16-items measure of perceived FoF, it ranges from a minimum of 16 (no fear/concerns of falling) to a maximum of 64 (strong concern about falling); iv) Barthel index (BI) to measure an individual’s ability to perform basic activities of daily living (ADLs) independently. Each activity is scored based on the level of assistance required, with a total possible score ranging from 0 to 100. Higher scores indicate greater independence, while lower scores suggest higher levels of dependency. Moreover, the neurologist administered the H&Y scale to classify PD patients according to their disability level.

### 2.4 Instrumental gait analysis

A skilled physiotherapist and a biomedical engineer assessed patients’ gait strides using the BTS Gaitlab (BTS Bioengineering, Milan, Italy) (Materia, 2024). During the instrumental GA assessment, patients were instructed to walk at a self-selected/comfortable speed to obtain the most accurate and representative data on their walking capabilities. This advanced GA system has fully integrated tools that enable objective, quantitative assessments for clinical use. Through instrumental analysis, clinicians and therapists gain an objective and quantitative view of changes in posture and gait, load imbalances, and muscle deficits often undetectable with standard clinical scales or tests.

The BTS Gaitlab system comprises.

- 8 infrared cameras (BTS SMART-DX)
- 4 force plates (BTS P-6000)
- 8 wireless EMG probes (BTS FREEEMG 1000)

**FIGURE 1:**
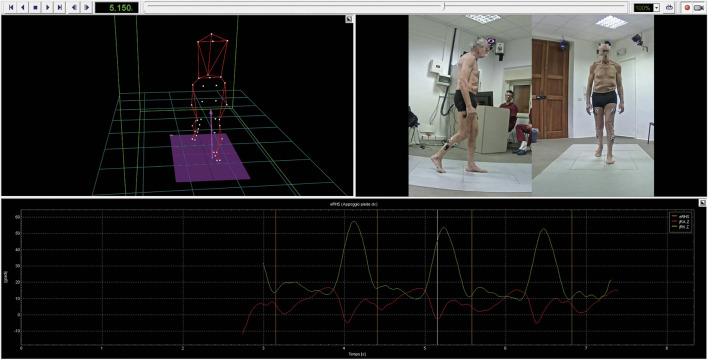
BTS SMART-Clinic software. The figure shows the data processing procedure for one of the participants. A 3D human body model, reconstructed using the optoelectronic system, is displayed along with the detected events and the synchronized video recordings from the gait trial.

The BTS SMART-Clinic software (Figure 1) includes libraries with scientifically validated protocols (Kadaba et al., 1989; Davis et al., 1991). In our study, we applied the “DAVIS Heel: multifactorial gait analysis” protocol, which provides quantitative, objective data on kinematics, kinetics, and associated muscle activity to assess gait functionality. This protocol is based on the “Newington marker set” from the Davis protocol (Davis et al., 1991) and requires measuring the participant’s anthropometric parameters, including weight, height, tibia length, femoral condyle distance, knee and ankle diameters, iliac crest distance, and pelvis thickness. The optoelectronic system captures the kinematic data by tracking marker positions on the patient’s body, calculating hip, knee, pelvis, trunk, and ankle joint angles in flexion-extension, abduction-adduction, and external-internal rotation. Events in the gait stride, such as initial ground contact and toe-off, are identified automatically through marker and force plate data, with manual verification by therapists.

The initial position (standing phase) is recorded by asking the patient to stand neutrally for 5 s. The patient then walks at a self-selected pace. After an average of six acquisitions (mean ± SD = 2.8 ± 0.9 strides per acquisition), baseline evaluation is complete. During analysis, EMG activity is also recorded through the eight wireless EMG probes (four on each limb), connected to the Smart Analyzer system (Version 1.10.469.0; BTS, Milan, Italy). In this study, EMG signals from the gastrocnemius lateralis (GL), tibialis anterior (TA), rectus femoris (RF), and semitendinosus (ST) muscles were evaluated following European guidelines for surface EMG (SENIAM) (Hermens et al., 2000), with skin prepared (cleaned and dried) before positioning bipolar surface electrodes aligned with muscle fiber orientation.

Normative data from healthy adults (40 participants: 28 males, 12 females, ages 18–40) (Kadaba et al., 1990) were used to compare the patients’ kinematic and kinetic, helping to identify gait dysfunction. Furthermore, data from BTS Gaitlab processed during offline analysis included:

#### 2.4.1 Kinematics

Spatio-temporal parameters, hip, knee, pelvis, trunk, and ankle joint angles in flexion-extension, abduction-adduction, and external-internal rotation measured during walking trials.

Spatio-temporal parameters.

• Spatio-temporal parameters were recorded during the walking trial. Normative data were obtained from BTS Gaitlab. Temporal aspects, including gait stride and stance duration (measured in seconds) as well as stance, swing, single and double support phases normalized to gait stride (%), were included alongside spatial parameters such as stride and step length, step width (m), and spatio-temporal parameters such as gait speed in (m/s). Spatio-temporal parameters were compared against the corresponding average gait feature for health people to identify gait impairments. Moreover, the gait profile score (Speciali et al., 2014), calculated as the Euclidean distance between the patient’s kinematic features and the corresponding normative features over the entire gait cycle, and the gait deviation index (Schwartz and Rozumalski, 2008), which indicates whether the subject’s gait features are statistically indistinguishable from those of the control group, were also evaluated. Additionally, the R_CIRC_ shape symmetry index (Equation 1) (Bonanno et al., 2024) was used to compare PD patients and healthy subjects in terms of joint rotation angles, reported with respect to the percentage of gait stride (%), quantifying gait dysfunction by estimating differences between pathological and normal gait patterns.

$$R_{CIRC}=\frac{C_{xy}}{\sqrt{\sum_{n=1}^{101}{x\left(n\right)}^{2}\sum_{n=1}^{101}{y\left(n\right)}^{2}}}$$ (1)

In which x is the waveform related to the PD subjects, while y corresponds to the average waveform of the healthy population. C_xy_ is the circular cross-correlation function at lag 0. R_CIRC_ ranges from −1 to 1 (i.e., identical amplitude profiles shape).

#### 2.4.2 Kinetics

Joint moments and powers at the hip, knee, and ankle joints:

• Kinetic results, including joint moments and powers normalized to the subject’s weight (Newton*meter/kg and Watt/kg) as well as ground reaction forces expressed as a percentage of body weight, were averaged across all gait strides for each participant. Additionally, the R_CIRC_ shape symmetry index (Equation 1) was calculated to compare joint moments, powers, and ground reaction forces between pathological and healthy groups.

#### 2.4.3 EMG

Muscle activation and co-activation patterns:

• Muscle activation signals were recorded with surface electrodes, capturing raw EMG signals (mV), which were filtered with a 20–450 Hz bandpass filter and rectified. The amplitude was normalized to the maximum of any channel and the time to the gait stride duration (% of gait stride). Signal amplitude, which is proportional to the force generated by the muscle, was analysed by calculating the co-contraction of agonist and antagonist muscles during gait enhances support, balance, propulsion, and movement efficiency. Estimating muscle co-contraction provides valuable insights into how a disorder may affect muscle coordination strategies. For this purpose, we used a co-contraction or co-activation index (CoAct) method based on (Equation 2), (Bonanno et al., 2024), where the normalized EMG of the antagonist muscle (norm EMG antago (t)) was the lower value and the normalized EMG of the agonist muscle (norm EMG ago (t)) was the higher value.

$$CoAct\left(t\right)=\frac{2\times norm\;EMG\left(t\right)antago}{\left(norm\;EMG\left(t\right)antago+norm\;EMG\left(t\right)ago\right)}\times 100$$ (2)

• In which the agonist-antagonist muscle pairs used for this analysis were TA-GL and RF-ST.

### 2.5 Statistical analysis

Kinematic and kinetic instrumental outcomes were compared to the normal values (Kadaba et al., 1990). The normal distribution of the samples for each parameter—including kinematic, kinetic and EMG—was investigated through Shapiro-Wilk test (MATLAB function swtest). According to the non-normality of most parameters, we chose a non-parametric analysis. The Wilcoxon rank sum test (MATLAB function ranksum) was used to compare overall pathological subjects’ parameters with healthy normative values. The same test was also applied to assess left-right sides differences. Data were analyzed both as averages between body sides and separately for each side, considering all gait strides of each participant.

We performed a clustering to partition the dataset into groups. To determine the optimal number of clusters, we performed clustering using the k-means algorithm (MATLAB function kmeans). We set a maximum of n = 20 clusters, iterating over possible cluster numbers from 2 to 20. We executed the k-means function on clinical scale evaluations, with 50 replicates to ensure stable convergence of the cluster centers. This generated the within-cluster sum of squared distances. To identify the ideal number of clusters, we evaluated the cumulative percentage of the explained variance. We selected the minimum number of clusters at which the explained variance reached or exceeded 90%, ensuring a balance between data representation accuracy and model simplicity.

Instrumental and clinical scales outcomes were analyzed with a nonparametric one-way ANOVA (Kruskal Wallis, MATLAB function kruskalwallis) and a LM (MATLAB function fitlm) because of the non-normal distribution of the kinematic data. The dependency of the response variable from the experimental factor clusters was tested with the Kruskal Wallis method, while the dependency from the experimental factor FES-I was tested with the LM in a *post hoc* analysis. The robust fitting type used for the LM was the “bisquare” weight function with the default tuning constant. All the analyses were implemented in Matlab (MATLAB (R2022a), Natick, Massachusetts: The MathWorks Inc.; 2022).

To assess whether our sample size was adequate for the analyses performed, we conducted two *post hoc* power analyses using G*Power (version 3.1.9.7). For the comparison between Parkinson’s patients and normative values, we used a t-test (Means: difference from constant, one sample case), with α = 0.05 and a total sample size of 69. For the comparison across clusters, we used an F test (ANOVA: fixed effects, omnibus, one-way), with α = 0.05, a total sample size of 69, and four groups. In both cases, the effect size was estimated from the data collected in our study, based on the variables compared. Specifically, for each analysis, we computed the effect size for each parameter separately using group means and standard deviations and then used the average value as input for the power calculation.

## 3 Results

**FIGURE 2:**
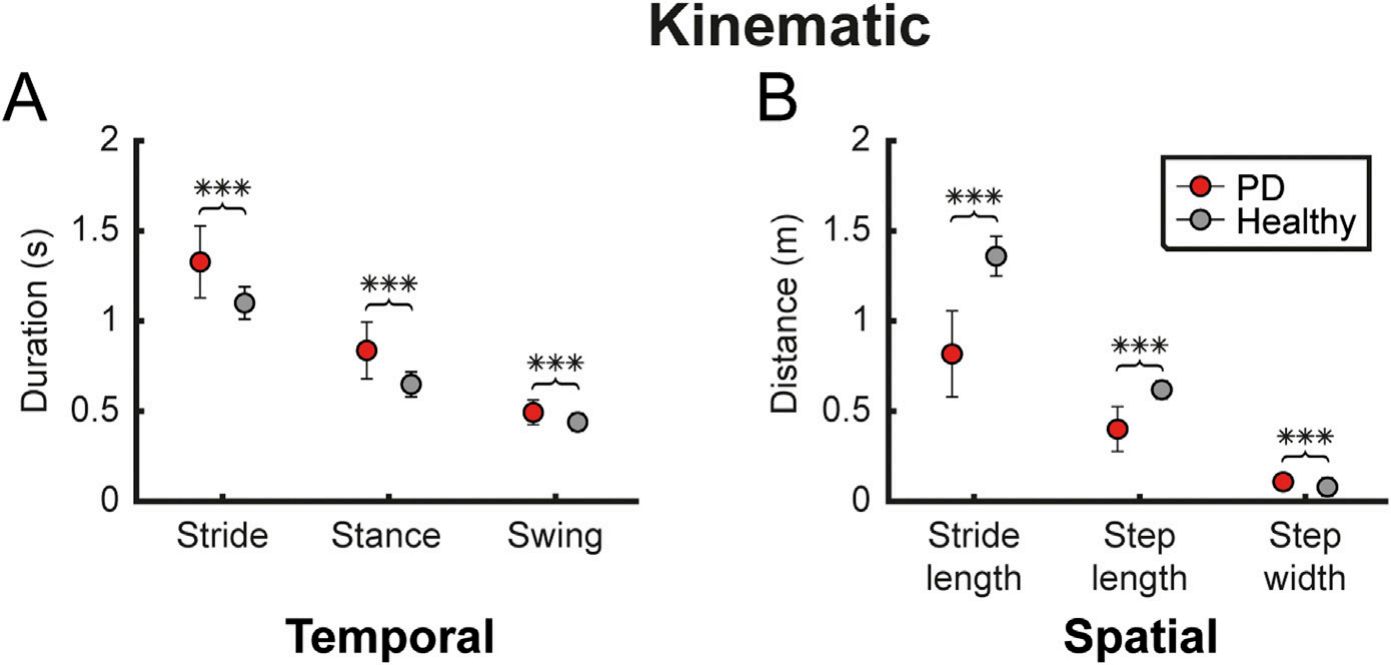
Kinematic gait parameters. The figure shows some of the temporal and spatial kinematic parameters, reported as mean ± standard deviation **(A)** Compares temporal parameters (gait stride, stance and swing duration) between participants with PD (red) and healthy subjects (grey) **(B)** Shows the same comparison for spatial parameters (stride length, step length and step width). Statistical difference significance, evaluated using Wilcoxon rank sum test, is reported as *** < 0.001, ** < 0.01 and * < 0.05.

Comparisons between participants’ instrumental and normative data revealed statistically significant differences in temporal and spatial kinematic parameters. Specifically, stride duration, stance duration, and swing duration average data were significantly higher than normative values (see Figure 2) showing a statistically significant difference (p < 0.001). The stance phase and double support phase also showed higher average values, whereas the swing phase and single support phase showed lower values compared with normative data showing a statistical difference (p < 0.001).

Both speed and cadence differed significantly from normative data (p < 0.001), with PD participants exhibiting lower values. Additionally, a reduced stride length and step length and an increased step width were observed, with a statistical difference (p < 0.001). The gait profile score was notably higher than normal, whereas the gait deviation index was lower, showing a statistical difference (p < 0.001).

The analysis of side-to-side symmetry revealed a statistically significant asymmetry in R_CIRC_ between left and right sides for both trunk obliquity (p < 0.001) and trunk rotation (p = 0.016) (see Supplementary Table S1).

The minimum number of clusters explaining at least 90% of the variance was four clusters, capturing 92% of the variance. Clusters were created based on clinical scales, presenting a gradient from the highest (Cluster 1) to the lowest motor functional state (Cluster 4) (See Table 2).

**TABLE 2:** Socio-demographic and clinical data of the clusters. Statistical difference between clusters was evaluated using nonparametric one-way ANOVA (Kruskal Wallis).

	Clusters				
	1	2	3	4	p-value
N subjects	21	12	21	15	
Gender					0.508
N male (%)	17 (81)	7 (58)	16 (76)	10 (67)	
N female (%)	4 (19)	5 (42)	5 (24)	5 (33)	
Age					
Mean ± SD	64 ± 8	70 ± 9	65 ± 11	69 ± 5	0.140
YSD					
Mean ± SD	4 ± 2	7 ± 4	7 ± 5	8 ± 3	**0.020**
H&Y					
Mean ± SD	2 ± 1	3 ± 1	3 ± 1	3 ± 1	**< 0.001**
BBS					
Mean ± SD	49 ± 3	43 ± 7	38 ± 5	33 ± 6	**< 0.001**
%	88 ± 6	77 ± 13	67 ± 10	59 ± 10	
TS					
Mean ± SD	25 ± 2	21 ± 3	18 ± 3	14 ± 2	**< 0.001**
%	88 ± 7	74 ± 12	64 ± 11	51 ± 7	
BI					
Mean ± SD	85 ± 12	84 ± 9	76 ± 8	61 ± 6	**< 0.001**
FES-I					
Mean ± SD	23 ± 5	46 ± 8	26 ± 5	48 ± 7	**< 0.001**
%	15 ± 10	61 ± 16	20 ± 11	66 ± 14	

Legend: YSD, years since disease; BBS, Berg Balance Scale; TS, Tinetti Scale; BI, Barthel Index; H&Y, Hoen & Yahr; FES-I, Falls Efficacy Scale–International. Significant p-values are reported in bold.

**FIGURE 3:**
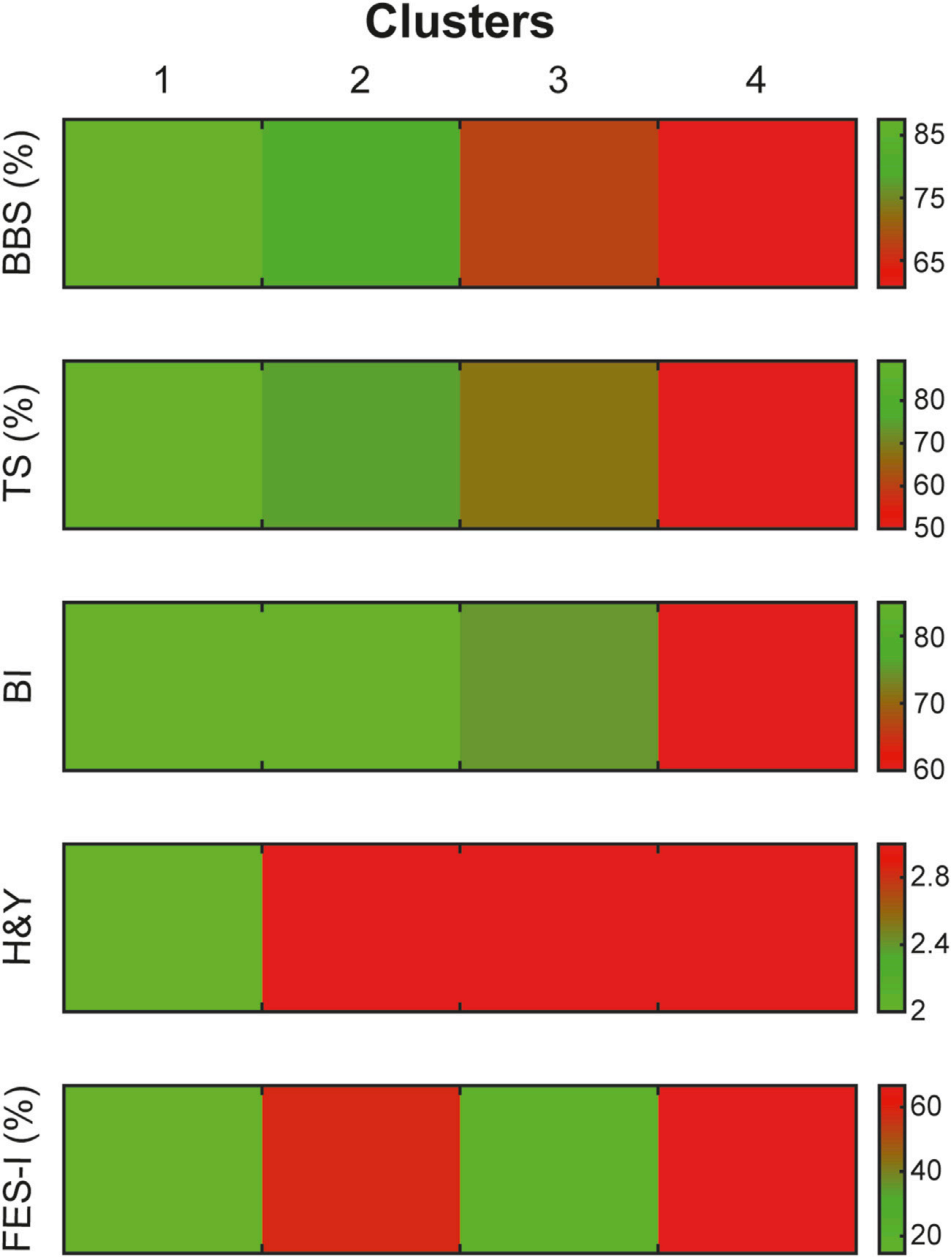
Distribution of clinical scale scores across identified clusters. The figure shows clustering results based on clinical scales, with a colour gradient representing severity levels on each scale. The gradient ranges from less severe (green) to more severe (red). This allows for an accurate visualization of severity patterns specific to each cluster and clinical scale.

This descending trend was observed across all the assessed scales (BBS, TS, BI, and H&Y), except for the FES-I scale, in which clusters 1 and 3 showed lower perceived FoF. Clusters two and 4 showed higher levels of FoF. Effectively, clusters categorized patients from least to most severe motor impairment, although they displayed distinct perceptions of FoF (see Figure 3).

**FIGURE 4:**
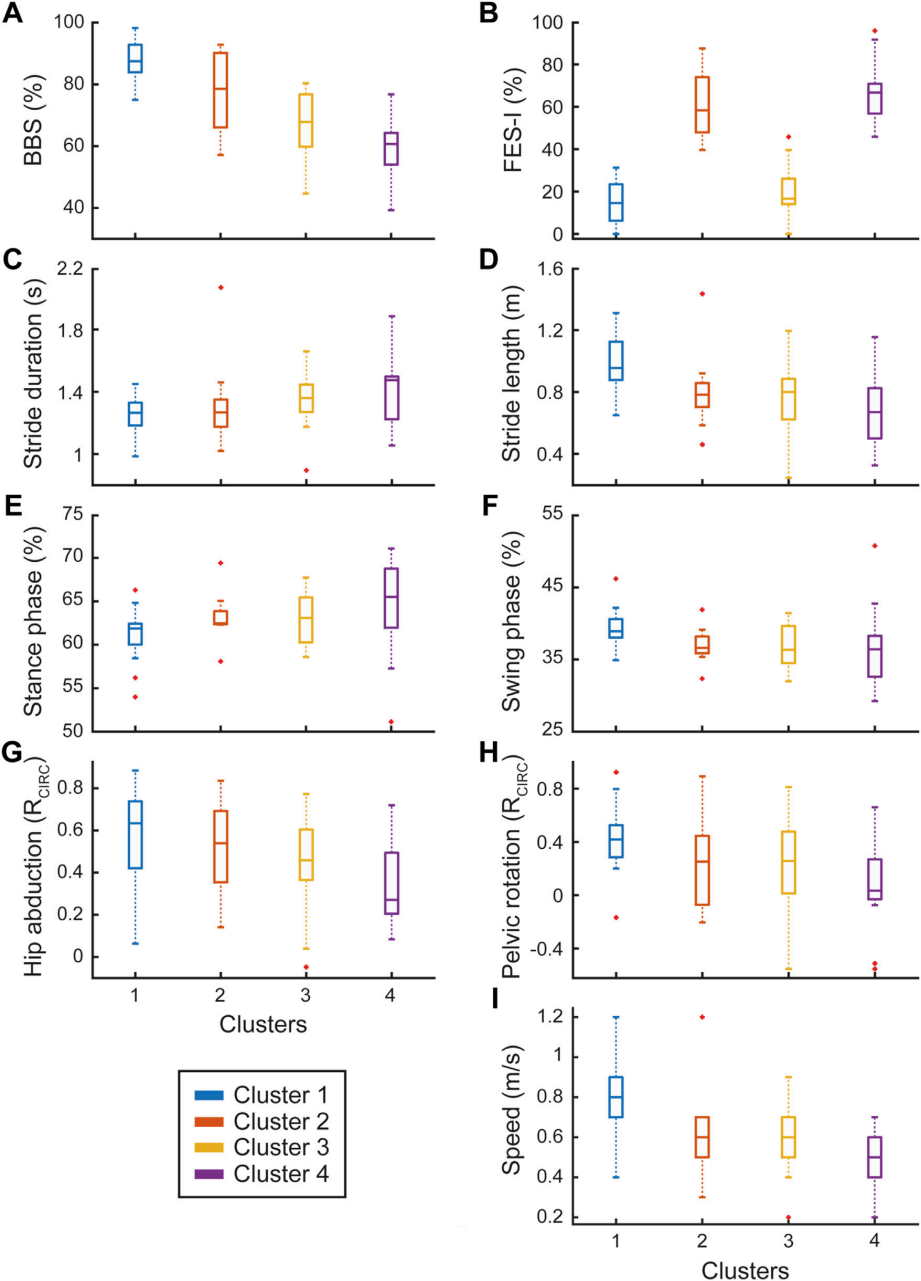
Motor clinical scales and instrumental biomechanical gait parameters. The left column shows instrumental parameters **(C,E,G)** with trends similar to BBS **(A)** while the right column includes those **(D,F,H,I)** following trends similar to FES-I **(B)**. Dependency of instrumental parameters from both clinical scales **(A,B)** was statistically confirmed. Each panel shows the box plots for the four identified clusters (represented by different colors), with red crosses indicating outlier values.

Statistical analysis indicated a significant difference between clusters and a significant effect of FES-I on the clinical scales: BBS (p < 0.001; *R* ^2^ = 0.29, p < 0.001, see Figure 4A), TS (p < 0.001; *R* ^2^ = 0.28, p < 0.001), BI (p < 0.001; *R* ^2^ = 0.16, p = 0.001), H&Y (p < 0.001; *R* ^2^ = 0.31, p < 0.001) and FES-I (p < 0.001; *R* ^2^ = 1, p < 0.001, see Figure 4B).

The ANOVA revealed significant differences among clusters across several instrumental parameters. In terms of temporal kinematics, we observed significant differences for stride duration (p = 0.049, see Figure 4C), stance duration (p = 0.013), stance phase percentage (p = 0.023, see Figure 4E), and single support phase percentage (p = 0.025). The spatial kinematic variable step width (p = 0.024) also showed significant cluster-based variation. Regarding results obtained from circular cross-correlation, the spatial kinematic hip abduction (p = 0.024, see Figure 4G) and the kinetics such as hip moment (p = 0.001), knee moment (p = 0.003), ankle moment (p = 0.004), hip power (p = 0.001), ankle power (p = 0.008), and medio-lateral force (p = 0.002) were significantly different across clusters. In EMG measurements, significant cluster differences were noted for the CoAct of the TA-GL muscles (p = 0.003).

For parameters influenced by both cluster and FES-I, we observed significant effects for temporal kinematics parameters including swing phase (p = 0.025; *R* ^2^ = 0.06, p = 0.049, see Figure 4F) and double support phase (p = 0.003; *R* ^2^ = 0.07, p = 0.046). Similarly, spatial kinematic parameters, such as stride length (p < 0.001; *R* ^2^ = 0.16, p = 0.001, see Figure 4D) and step length (p < 0.001; *R* ^2^ = 0.15, p = 0.001) showed significant effects. The spatiotemporal kinematic variable, velocity, was significantly influenced by both cluster and FES-I (p < 0.001; *R* ^2^ = 0.13, p = 0.002, see Figure 4I). Joint angles as well as kinetic measurements also demonstrated dual significance for the circular cross-correlation of pelvic rotation (p = 0.032; *R* ^2^ = 0.11, p = 0.006, see Figure 4H), knee flexion-extension (p = 0.007; *R* ^2^ = 0.09, p = 0.015), anterior-posterior force (p < 0.001; *R* ^2^ = 0.13, p = 0.004), and vertical force (p < 0.001; *R* ^2^ = 0.14, p = 0.002).

In line with our findings, the dependency of some instrumental biomechanical gait parameters on motor clinical scales was confirmed, as illustrated in Figure 4 (left column), in which the boxplots of the four clusters follow the BBS’s trend. On the other hand, the parameters in the right column followed the FES-I’s trend among clusters.

Lastly, parameters affected solely by the FES-I score included EMG CoAct of the RF-ST muscles (*R* ^2^ = 0.10, p = 0.01).

To sum up, we provided a detailed table reporting mean and SD values for each cluster across all the evaluated parameters and the relative statistical comparison results (see Table 3). We also reported the mean values for each instrumental parameter (columns) and cluster (rows) in the matrix shown in Supplementary Figure S1. The figure shows the instrumental results for each cluster and parameter, with a color gradient representing the relative performance on each scale. The gradient ranges from better performance (green) to worse performance (red), allowing for a clear visualization of how each cluster performs across the different instrumental parameters.

**TABLE 3:** Instrumental data across clusters. The table reports instrumental variables (mean ± SD) for the four identified clusters. Statistical differences between clusters were assessed using nonparametric one-way ANOVA (Kruskal Wallis test). The influence of the FES-I score was evaluated through a LM.

		Gait parameters	Cluster 1 Mean ± SD	Cluster 2 Mean ± SD	Cluster 3 Mean ± SD	Cluster 4 Mean ± SD	Cluster p-value	LM FES-I p-value (R^2^)
Kinematic	Temporal	Stride duration (s)	1.257 ± 0.123	1.31 ± 0.274	1.348 ± 0.159	1.415 ± 0.238	**0.049**	0.097 (0.04)
		Stance duration (s)	0.769 ± 0.091	0.833 ± 0.213	0.85 ± 0.111	0.919 ± 0.198	**0.013**	0.096 (0.04)
		Swing duration (s)	0.491 ± 0.048	0.481 ± 0.074	0.492 ± 0.065	0.511 ± 0.081	0.501	0.836 (0.01)
		Stance phase (%)	61.232 ± 2.829	63.173 ± 2.578	63.096 ± 2.876	64.569 ± 5.402	**0.023**	0.063 (0.05)
		Swing phase (%)	39.15 ± 2.544	37.013 ± 2.338	36.768 ± 2.834	36.684 ± 5.423	**0.025**	**0.049** (0.06)
		Single stance phase (%)	39.15 ± 2.541	37.039 ± 2.352	36.825 ± 2.833	36.838 ± 5.807	**0.025**	0.052 (0.06)
		Double stance phase (%)	12.106 ± 3.421	13.505 ± 2.387	14.052 ± 3.506	16.715 ± 5.268	**0.003**	**0.046** (0.07)
		Mean speed (m/s)	0.8 ± 0.161	0.625 ± 0.222	0.571 ± 0.187	0.487 ± 0.155	**< 0.001**	**0.002** (0.13)
		Cadence (steps/min)	96.633 ± 9.767	94.895 ± 15.657	91.333 ± 12.791	87.704 ± 14.878	0.069	0.093 (0.04)
	Spatial	Stride length (m)	0.983 ± 0.161	0.804 ± 0.236	0.763 ± 0.234	0.676 ± 0.227	**< 0.001**	**0.001** (0.16)
		Step length (m)	0.487 ± 0.081	0.402 ± 0.117	0.382 ± 0.113	0.309 ± 0.12	**< 0.001**	**0.001** (0.15)
		Step width (m)	0.115 ± 0.031	0.094 ± 0.027	0.095 ± 0.043	0.129 ± 0.042	**0.024**	0.595 (0.01)
		Gait profile score (deg)	8.271 ± 2.509	8.8 ± 1.966	10.098 ± 2.936	9.65 ± 2.088	0.118	0.307 (0.02)
		Gait deviation index	87.83 ± 14.542	84.71 ± 10.689	86.146 ± 14.953	80.887 ± 9.838	0.449	0.169 (0.03)
		Pelvic obliquity (R_CIRC_)	0.087 ± 0.366	0.142 ± 0.298	0.04 ± 0.331	-0.101 ± 0.367	0.278	0.369 (0.01)
		Pelvic tilt (R_CIRC_)	0.531 ± 0.848	0.666 ± 0.75	0.994 ± 0.013	0.596 ± 0.82	0.272	0.601 (0.56)
		Pelvic rotation (R_CIRC_)	0.418 ± 0.221	0.235 ± 0.331	0.267 ± 0.356	0.084 ± 0.341	**0.032**	**0.006** (0.11)
		Hip abduction-adduction (R_CIRC_)	0.574 ± 0.228	0.52 ± 0.222	0.452 ± 0.217	0.356 ± 0.202	**0.024**	0.059 (0.05)
		Hip flexion-extension (R_CIRC_)	0.831 ± 0.274	0.87 ± 0.288	0.931 ± 0.054	0.854 ± 0.232	0.251	0.762 (0.01)
		Hip rotation (R_CIRC_)	0.233 ± 0.255	0.316 ± 0.245	0.213 ± 0.258	0.358 ± 0.317	0.246	0.134 (0.03)
		Knee flexion-extension (R_CIRC_)	0.965 ± 0.036	0.947 ± 0.051	0.936 ± 0.046	0.916 ± 0.063	**0.007**	**0.015** (0.09)
		Ankle dorsiflexion-plantiflexion (R_CIRC_)	0.617 ± 0.196	0.525 ± 0.253	0.461 ± 0.262	0.438 ± 0.244	0.144	0.401 (0.01)
		Foot progression (R_CIRC_)	0.919 ± 0.165	0.95 ± 0.039	0.858 ± 0.237	0.895 ± 0.145	0.106	0.110 (0.24)
		Trunk tilt (R_CIRC_)	0.522 ± 0.824	0.42 ± 0.838	0.353 ± 0.911	0.704 ± 0.687	0.176	0.148 (0.70)
		Trunk obliquity (R_CIRC_)	0.219 ± 0.166	0.169 ± 0.207	0.189 ± 0.218	0.212 ± 0.223	0.666	0.385 (0.08)
		Trunk rotation (R_CIRC_)	0.425 ± 0.2	0.434 ± 0.309	0.365 ± 0.259	0.473 ± 0.227	0.715	0.537 (0.01)
		Knee Varus-valgus (R_CIRC_)	0.474 ± 0.48	0.446 ± 0.514	0.51 ± 0.404	0.602 ± 0.28	0.972	0.721 (0)
		Knee rotation (R_CIRC_)	0.157 ± 0.513	0.201 ± 0.654	0.118 ± 0.477	0.26 ± 0.459	0.762	0.475 (0.01)
Kinetic	Joint moments	Hip (R_CIRC_)	0.844 ± 0.104	0.783 ± 0.141	0.516 ± 0.381	0.645 ± 0.262	**0.001**	0.322 (0.02)
		Knee (R_CIRC_)	0.473 ± 0.201	0.375 ± 0.292	0.225 ± 0.197	0.286 ± 0.198	**0.003**	0.305 (0.02)
		Ankle (R_CIRC_)	0.966 ± 0.029	0.944 ± 0.071	0.92 ± 0.043	0.936 ± 0.05	**0.004**	0.646 (0)
	Joint powers	Hip (R_CIRC_)	0.688 ± 0.175	0.629 ± 0.17	0.375 ± 0.309	0.438 ± 0.28	**0.001**	0.224 (0.02)
		Knee (R_CIRC_)	0.377 ± 0.326	0.21 ± 0.497	0.118 ± 0.404	0.28 ± 0.342	0.304	0.607 (0)
		Ankle (R_CIRC_)	0.692 ± 0.233	0.554 ± 0.215	0.489 ± 0.362	0.389 ± 0.239	**0.008**	0.065 (0.05)
	Ground reaction forces	Anterior-posterior (R_CIRC_)	0.934 ± 0.039	0.903 ± 0.046	0.801 ± 0.249	0.721 ± 0.306	**< 0.001**	**0.004** (0.13)
		Medio-lateral (R_CIRC_)	0.94 ± 0.025	0.936 ± 0.02	0.918 ± 0.021	0.906 ± 0.037	**0.002**	0.190 (0.03)
		Vertical (R_CIRC_)	0.982 ± 0.007	0.971 ± 0.017	0.97 ± 0.012	0.96 ± 0.018	**< 0.001**	**0.002** (0.14)
EMG	Agonist-antagonist co-activation	TA-GL (CoAct)	47.82 ± 18.767	31.186 ± 8.918	32.594 ± 13.852	49.237 ± 20.092	**0.003**	0.801 (0.01)
		RF-ST (CoAct)	25.008 ± 12.592	36.898 ± 23.835	25.046 ± 12.855	33.224 ± 13.436	0.248	**0.010** (0.10)

Legend: LM, linear model; SD, standard deviation; *R* ^2^, coefficient of determination; deg, degree; R_CIRC_, shape symmetry index; TA-GL, Tibialis anterior-gastrocnemius lateralis; RF-ST, Rectus femoris-semitendinosus; CoAct, co-activation index. Significant p-values are reported in bold.

The results from the *post hoc* power analysis indicated that our sample size was sufficient to support the main comparisons. We conducted two *post hoc* power analyses to assess this formally. For the comparison between PD patients and normative values, the power analysis using the observed average effect size (Cohen’s d = 0.5) indicated a high statistical power (1–β = 0.99). For the comparison across the four Parkinson clusters, the observed average effect size (Cohen’s f = 0.4) yielded a power of 0.78, indicating sufficient sensitivity for detecting medium-to-large differences among groups.

## 4 Discussion

In this study, we highlight the importance of complementing clinical assessments with instrumental GA. While comparing gait biomechanics between PD patients and healthy individuals is not novel in itself, our study enhances and strengthens existing literature by providing a comprehensive biomechanical assessment, including kinematics, kinetics, and EMG analysis, as detailed in (see Supplementary Table S1). Notably, we also report joint angular measurements, which are underrepresented in the literature, as noted by Zanardi et al. in their systematic review with meta-analysis.

Additionally, the clustering analysis, which classified patients based on their clinical scores, revealed that certain instrumental GA parameters, such as stride length, swing phase, pelvic rotation, and gait speed, do not follow the same pattern as clinical scales, highlighting the importance of objective gait assessment. Similarly, Russo et al. conducted a cluster analysis on spatiotemporal parameters extracted from an optoelectronic device in a sample of people with PD (Russo et al., 2023). However, our study goes further by incorporating kinematic angular, kinetic, and EMG parameters alongside clinical scales, adding a novel dimension to our findings. Furthermore, we identified specific dysfunctional gait patterns in individuals with PD who experience higher levels FoF, providing valuable insights into the prevention of potential falls in this patient population.

Consequently, these findings can provide new perspectives to better guide rehabilitation strategies for people with PD, according to their specific needs.

### 4.1 Instrumental and biomechanical gait parameters

In this observational study, we first aimed to characterize the biomechanical pattern of gait in a cohort of PD patients compared to normal values. Our results suggest that PD patients had significant differences in the kinematic parameters of gait. Indeed, PD patients showed higher temporal (stride, stance and swing duration) parameters than normative values. Conversely, spatial parameters such as stride length and step length were notably reduced in PD patients, compared to normative values. These findings align with previous research (Pistacchi et al., 2017; Zanardi et al., 2021; Russo et al., 2025) indicating that PD-related gait alterations commonly involve prolonged time spent in each phase of the gait cycle, accompanied by shorter step and stride lengths. According to Peppe et al., patients with PD exhibit an increased flexion in the hip, knee, and ankle, both in standing and walking positions, compared to control subjects. Moreover, these authors (Peppe et al., 2007) found that following the motor rehabilitation program, the joint angle values in PD patients, except for the knees, were closer to those observed in the control group. Regarding kinematic results, we also found that people with PD exhibited higher gait profile score compared to normative values. Gait profile score is a single score that represents the overall deviation of gait kinematics from normative data, providing a quantitative assessment of the gait profile. This score is derived from the analysis of nine kinematic variables, evaluated on both sides of the body (Speciali et al., 2014). This result is in line with findings of other studies showing an altered gait profile, possibly explained by the variation found in some of the gait profile score domains (including knee flexion/extension and pelvic obliquity). These angular changes in gait are commonly found in PD and are justified by the reduction in range of motion of lower limbs and difficulty in regulating and coordinating movements during gait (Speciali et al., 2014; Pistacchi et al., 2017; Callais Franco do Nascimento et al., 2021). In addition, we found that the gait deviation index was lower in people with PD compared to normative values. This index is a multivariate measure of overall gait pathology based on 15 gait features derived from 3D kinematic data, offering a clear, comprehensive, and clinically relevant measure of overall gait function (Schwartz and Rozumalski, 2008). In the field of PD, only two studies have explored the role of the Gait deviation index in Parkinsonian gait. Specifically, Galli et al., suggested gait deviation index was more useful for assessing the impact of levodopa treatment on gait rather than for identifying the severity of gait pathology in PD patients (Galli et al., 2012). Other findings were reported by Speciali et al., who stated that this index effectively captures gait alterations during dual-task exercises. Additionally, we found a significant difference between the left and right side in the trunk obliquity and trunk rotation R_CIRC_, suggesting a potential musculoskeletal asymmetry between the two sides. According to Cano-de-la-Cuerda et al., trunk alterations may result from axial muscle stiffness. Patients may exhibit a reduced joint range of motion due to excessively high muscle tone, which restricts full articulation and leads to asymmetries between the two sides.

In this vein, it is noteworthy that the use of instrumental GA can provide objective outcome measures for a rehabilitation program. It can also contribute to give additional information on specific dysfunctional gait patterns that could aid in understanding the complex pathophysiology of PD.

### 4.2 Biomechanical gait and clinical differences among clusters

Secondly, we aimed to investigate biomechanical gait differences among PD patients by clustering them into four groups based on clinical rating scales (H&Y, BBS, TS, BI, and FES-I) and analysing variations in instrumental biomechanical data across these clusters. We found statistically significant differences among clusters in kinetic gait parameters, such as hip, knee, and ankle moments, as well as hip and knee power. These differences could be related to disease progression, as patients with more severe impairment (such as those in Cluster 4) may have developed compensatory mechanisms, such as a hip-driven strategy to compensate for reduced ankle and knee function. This hypothesis is further supported by the observed CoAct of the RF-ST muscles, which may indicate increased recruitment of proximal muscles rather than distal ones, such as TA-GL (Islam et al., 2020). Notably, patients with higher levels of disability may need to reduce their knee power and moment to minimize lower limb instability, which could increase the risk of falls. Additionally, the typical muscle rigidity and bradykinesia in individuals with PD may lead to a reduced range of motion, consequently diminishing lower limb power. This reduction appears to be closely related to the severity of impairment within each cluster. Furthermore, we observed a statistical difference among clusters regarding medio-lateral force. In particular, Cluster 4 showed a reduction in medio-lateral force, which may indicate a more cautious gait pattern aimed at minimizing balance perturbations. This reduced medio-lateral force could reflect compensatory strategy to minimize balance perturbations, given the increased postural instability in patients with severe PD. This aligns with previous research (Kawami et al., 2022) indicating that PD patients with medio-lateral balance impairments demonstrate reduced variability in medio-lateral center of mass movements, potentially as a response to increased postural sway during gait. While this strategy may initially help stabilize gait, it may also lead to a narrower movement area, reducing the ability to make necessary postural adjustments and ultimately increasing fall risk. In this vein, rehabilitation interventions should aim to enhance medio-lateral stability and optimize stance width to improve balance control and prevent falls in patients with advanced PD (Kawami et al., 2022).

Interestingly, according to our cluster analysis, we found that PD patients can be grouped into two main categories, those with high levels of FoF and those with low levels of FoF independent of their motor impairment. FoF is a common symptom in patients with PD, and it is considered one of the most stressful physical symptoms (Jonasson et al., 2018). In addition, FoF was found to be a predictor of future falls and near falls already in mild PD and is negatively associated with participation and health related as well as overall quality of life. In our study, we found that PD patients with high levels of FoF had specific dysfunctional gait patterns that were different from those with low levels of FoF. In line with our results, some authors suggested that gait speed and stride lengths were poorer in people with a high level of FoF (Bryant et al., 2014), while Uhlig et al., (Uhlig and Prell, 2023a), found that only gait speed was associated with FoF.

This fear, along with decreased confidence in one’s ability to perform daily activities, can contribute to or worsen cautious walking. In PD, reduced gait speed—linked to a more cautious gait and FoF—can be strongly associated with previous fall incidents (Mak and Pang, 2009; Kataoka et al., 2011; Liu et al., 2022; Uhlig and Prell, 2023a). However, we did not analyse this aspect, but in future studies this aspect should be further investigated, correlating previous fall incidents with the FoF and biomechanical parameters.

A cautious gait, which is also commonly seen in healthy older adults (Uhlig and Prell, 2023b), involves slower gait speed, shorter step length, and lower toe clearance, allowing the person to stay “as close as possible” to the floor for further stability. Compared to a previous study (Bryant et al., 2014) in literature, our findings indicate that in individuals with high levels of FoF, not only gait speed is reduced, but we also observed an increase in cadence and a decrease in stride length. These results suggest that PD patients who experience higher FoF tend to adopt shorter, more controlled steps, characterized by reduced speed but an increased step frequency. This altered gait pattern could potentially create a vicious cycle, leading to greater energy expenditure at the muscular level. A recent meta-analysis suggested that PD patients compared to healthy controls manifest a reduced muscle activity of gastrocnemius medial and higher activity of TA, accompanied by a higher co-contraction of these ankle muscles during gait. These alterations can influence adequate transfer weight in preparation for stepping and it can reflect in a higher metabolic cost of walking (Zanardi et al., 2021). However, we found that patients with higher FoF manifested an increased CoAct of RF-ST that was significantly correlated with FES-I score. From a biomechanical point of view, EMG co-contraction patterns could have a compensatory role in increased proximal muscle activity (e.g., RF-ST) in response to reduced distal muscle function. Enhanced quadriceps activation during the stance phase promotes greater knee extension, improving joint stability in single stance and potentially compensating for reduced ankle stability (Islam et al., 2020). Similarly, increased hamstring activity during the swing phase enhances hip extension and knee flexion, partially substituting for the foot placement and initial loading functions typically performed by distal ankle muscles. However, this increased muscle activity also raises metabolic demand, which may contribute to reduced walking speed and mobility. Moreover, it is worth noting that muscle rigidity is a cardinal symptom of PD, as defined by the International Parkinson and Movement Disorder Society. Rigidity in PD is characterized by a velocity-independent increase in muscle tone, assessed through passive muscle stretch at specific joints. Importantly, rigidity and postural instability, two hallmark motor features of PD, are likely to shape EMG patterns during gait. Baradaran et al. demonstrated that rigidity is linked to altered cortical/subcortical connectivity, including changes in the supplementary motor area and putamen, along with increased motor cortex excitability (Baradaran et al., 2013). A potential consequence of this disrupted neural control is heightened agonist/antagonist co-contraction and reduced selective muscle recruitment, which may contribute to gait dynamic instability. This instability, defined as difficulty transitioning between gait phases, can manifest as prolonged double-support time and increased stride time variability. Other elements that could have an influence of biomechanical gait parameters are cognitive and psychological factors, like anxiety (Zhang et al., 2024). While assessing cognitive and psychological status was beyond the scope of our study, future research integrating a comprehensive biomechanical assessment with motor and cognitive/psychological evaluations could offer deeper insights into gait characteristics in individuals with PD.

Furthermore, we found that pelvic rotation and knee flexion extension were reduced when compared to normative values, especially in PD patients with high levels of FoF. This finding could be explained by the fact that patients with high FoF tend to adopt a more cautious gait to avoid falling, limiting the range of motion in their knees and pelvis. What is more, FoF could increase muscle stiffness, as we found within the CoAct of lower limb muscles, further reducing the motion flexibility of the knee and pelvis. In addition, the reduced knee flexion-extension and pelvic rotation may indicate a diminished ability to generate joint power in the hip and knee, as we found in Cluster 4. This limitation in movement could be associated with decreased gait propulsion and coordination, which are further exacerbated by muscle rigidity and difficulty executing broad and rapid movements (Paul et al., 2012). Another aspect we investigated involves ground reaction forces, specifically the anterior-posterior and vertical forces exerted by the ground on the body during movement (Chockalingam et al., 2016; Alam et al., 2017). We observed that PD patients with high levels of FoF tend to exhibit ground reaction forces in the anterior-posterior and vertical directions that deviate more significantly from normal values during gait, which is in line with the current literature (Russo et al., 2025). This finding could suggest that they may exert dysfunctional forces in these directions, possibly because high FoF leads them to move more cautiously to avoid falling. In this context, Oh et al. demonstrated that people with PD exhibited a lower peak in both the vertical and anterior-posterior ground reaction forces during propulsion (Oh et al., 2020), which aligns with our observation of altered ground reaction forces patterns in PD patients with high levels of FoF.

### 4.3 Strengths, limitations, and future perspectives

In this study, we highlighted the fundamental role of instrumental GA and clinical motor assessment, suggesting that the combined use of both these tools can be useful to assess quantitatively and objectively motor and biomechanical features of gait in PD patients. However, one of our strengths is that we analyzed several biomechanical parameters, including not only kinematics, but also kinetics and electrophysiological data, which are often neglected in clinical contexts.

Another strength of our study is the use of an optoelectronic MoCap in addition to force platforms and EMG probes to analyze gait biomechanics. In this sense, only a few authors have used this comprehensive GA laboratory (Pistacchi et al., 2017; Russo et al., 2023), which is considered the gold standard for the analysis of biomechanics of human movement (Ceseracciu et al., 2014). Despite this important advantage of this type of MoCap, it does not allow real-world movements detection, as wearable devices can do. For instance, wearable devices can be used outside the laboratory setting giving a relatively realistic representation of daily life relevant mobility aspects (Scott et al., 2022), providing kinematic gait data such as step length, stride width (Rossanigo et al., 2023). However, the amount of data that can be gathered in a laboratory is generally greater than that obtained from wearable sensors, unless multiple systems are used synchronously (e.g., EMG, a suit with multiple IMUs, insoles for plantar pressure recording). Nevertheless, we chose to use a controlled recording environment to ensure both the standardization of the acquisition protocol and safety reasons. In comparison with other previous studies (Pistacchi et al., 2017; Schniepp et al., 2021; Zanardi et al., 2021), we used methods to classify PD patients according to clinical assessment in order to evaluate the impact of the level of impairment on the instrumental gait parameters, by using clustering that aimed to discover naturally occurring patterns or groupings within the data. This type of analysis was also performed by Russo et al., in which they extracted two clusters based on spatial-temporal gait parameters from a sample of people with PD. However, they did not include kinetic factors and EMG signals. In this sense, our work expanded and enriched the literature in this field, suggesting that a comprehensive instrumental and clinical assessment is fundamental to address personalized patients’ needs for rehabilitation.

In addition, to analyze gait patterns across gait stride, we used methods, like the circular cross-correlation. On the other hand, to describe the energetic costs related to dysfunctional of gait, we performed methods to quantify the co-contraction between antagonist and agonist muscles.

Our study has some limitations that need to be acknowledged. For instance, a generalization of our results is impossible due to the small number of PD patients involved in our sample. This is why, in some biomechanical gait parameters we observed only a trend but not a statistical significance. However, the sample size aligns with similar observational studies in the field (Zanardi et al., 2021). Moreover, *post hoc* power analyses confirmed that the collected sample size was sufficient to detect medium to large effects in both the comparison with normative values and the cluster-based analysis, supporting the reliability of the reported findings. Nevertheless, in the cluster-based comparison, smaller differences between groups may have gone undetected due to slightly reduced statistical power, and future studies with larger samples may help clarify subtler distinctions.

Another limitation is the lack of additional clinical data, such as history of previous falls, cognitive functioning, and/or pharmacological dosages, which could have strengthened our results. Moreover, a limitation of this study is the absence of a formal priori power analysis, as the dataset was derived from real-life clinical evaluations conducted during routine practice. Although we have not collected normative data in our own gait analysis laboratory, which could lead to potential biases related to the recording procedures, the normative datasets used in the literature (Kadaba et al., 1990) use instrumentation with similar accuracy and comparable walking distances. In addition, a potential limitation of this study is the lack of an explicit assessment of multicollinearity among the analyzed gait parameters. Given the high correlation that may exist between spatiotemporal, kinematic, and kinetic variables, future studies should incorporate specific statistical approaches to address this issue and minimize the risk of redundancy or misinterpretation of results.

In future studies, the level of FoF should be addressed during the rehabilitation interventions. In fact, it is important to note that the FoF is not merely a perceptual or psychological factor, but it also has a clear impact on walking performance, including kinematics, kinetics and electrophysiological aspects. For example, cognitive and motor training based on VR technologies that add multisensorial stimulation, and feedback can be useful to reduce FoF, since they can simulate different scenarios in a safe and controlled environment (Bonanno et al., 2024; Impellizzeri et al., 2024).

## 5 Conclusion

Our results highlight that PD affects a wide range of biomechanical gait parameters, including the kinematic, kinetic, and electrophysiological aspects. These gait abnormalities cannot be detected by clinical scales especially in the early stages, as they primarily assess the gross motor functions. Furthermore, we found that patients with higher levels of FoF exhibited distinct biomechanical alterations compared to those with lower levels of FoF, independent of impairment severity, gait and balance dysfunction, and overall independence. However, the question remains open as to whether PD patients tend to manifest FoF due to the motor alterations caused by the disease, or if it is the FoF following the onset of the disease that leads to these alterations. Further larger sample longitudinal studies are needed to better understand gait abnormalities in PD patients and then tailor personalized rehabilitation plans.

## Data Availability

The raw data supporting the conclusions of this article will be made available by the authors, without undue reservation.
