# Supplementary material for: Beyond clinical scales: an observational study on instrumental gait analysis and biomechanical patterns in patients with Parkinson’s disease
Source: Front Bioeng Biotechnol. 2025 Apr 30;13:1541240. doi: 10.3389/fbioe.2025.1541240 (PMC12075126; doi:10.3389/fbioe.2025.1541240)
Supplement: Supplementary file 1 [file DataSheet1.pdf]

Supplementary Material

1 Supplementary Figures and Tables

1.1 Supplementary Figure

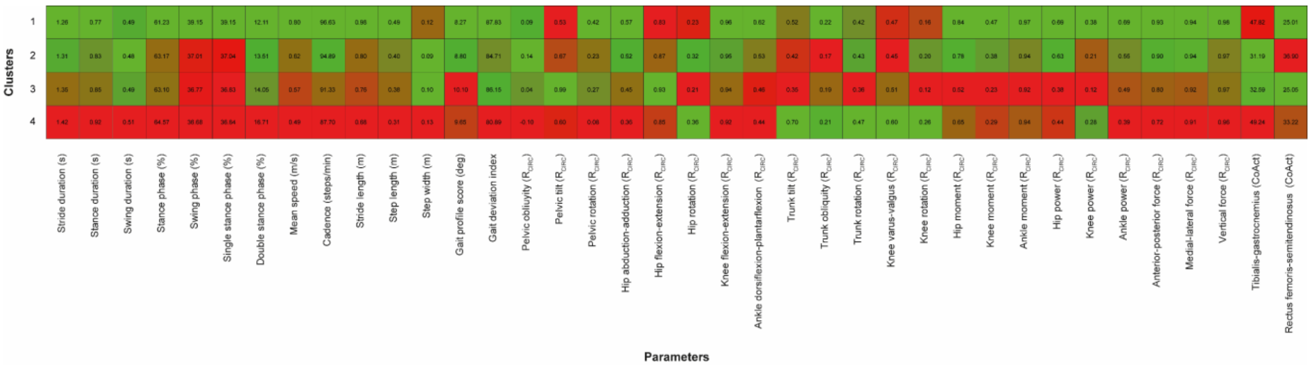

Supplementary figure 1. Heatmap of mean instrumental values across the four identified clusters. Figure shows a heatmap in which rows represent clusters and columns correspond to the different instrumental parameters. Cell colors reflect the relative performance of each cluster for each parameter, with a gradient from green (best performance) to red (worst performance).

1.2 Supplementary Table

Supplementary table 1. Instrumental parameters and left-right sides comparison. Table shows instrumental parameters (mean ± SD) reported separately for the left side (LS), right side (RS), and their mean (LS & RS). The table also includes p-values from the Wilcoxon rank-sum test used to assess statistical differences between LS and RS.

		Gait parameters	LS VS RS<br>p-value	LS<br>Mean ± SD	RS<br>Mean ± SD	LS & RS<br>Mean ± SD
Kinematic	Temporal	**Stride duration (s)**	0.831	1.333 ± 0.204	1.323 ± 0.196	1.328 ± 0.199
		**Stance duration (s)**	0.969	0.837 ± 0.162	0.838 ± 0.06	0.837 ± 0.154
		**Swing duration (s)**	0.903	0.495 ± 0.037	0.493 ± 0.073	0.494 ± 0.065
		**Stance phase (%)**	0.848	62.686 ± 4.334	63.039 ± 3.793	62.862 ± 4.061
		**Swing phase (%)**	0.818	37.513 ± 4.281	37.522 ± 3.433	37.517 ± 3.866
		**Single stance phase (%)**	0.805	37.322 ± 3.391	37.823 ± 5.112	37.573 ± 4.329
		**Double stance phase (%)**	0.846	14.085 ± 5.676	13.801 ± 3.77	13.943 ± 4.803
		**Mean speed (m/s)**	1	0.632 ± 0.212		
		**Cadence (steps/min)**	1	92.777 ± 13.109		
	Spatial	**Stride length (m)**	0.956	0.82 ± 0.24	0.816 ± 0.24	0.818 ± 0.239
		**Step length (m)**	0.67	0.4 ± 0.126	0.403 ± 0.124	0.401 ± 0.125
		**Step width (m)**	1	0.109 ± 0.039		0.109 ± 0.039
		**Gait profile score (deg)**	0.486	9.435 ± 3.46	9.003 ± 2.607	9.219 ± 3.06
		**Gait deviation index**	0.543	84.803 ± 15.229	85.728 ± 13.759	85.266 ± 14.467
		**Pelvic obliquity (R_{CIRC})**	0.667	0.06 ± 0.471	0.023 ± 0.47	0.041 ± 0.469
		**Pelvic tilt (R_{CIRC})**	0.871	0.71 ± 0.691	0.709 ± 0.693	0.709 ± 0.69
		**Pelvic rotation (R_{CIRC})**	0.922	0.262 ± 0.34	0.273 ± 0.326	0.267 ± 0.332
		**Hip abduction-adduction (R_{CIRC})**	0.545	0.471 ± 0.29	0.471 ± 0.313	0.48 ± 0.301
		**Hip flexion-extension (R_{CIRC})**	0.563	0.872 ± 0.217	0.875 ± 0.238	0.874 ± 0.227
		**Hip rotation (R_{CIRC})**	0.242	0.23 ± 0.331	0.307 ± 0.307	0.269 ± 0.32

		**Knee flexion-extension (R_{CIRC})**	0.838	0.945 ± 0.052	0.939 ± 0.067	0.942 ± 0.06
		**Ankle dorsiflexion-plantiflexion (R_{CIRC})**	0.815	0.527 ± 0.254	0.502 ± 0.289	0.515 ± 0.271
		**Trunk tilt (R_{CIRC})**	0.805	0.489 ± 0.821	0.496 ± 0.819	0.492 ± 0.817
		**Trunk obliquity (R_{CIRC})**	**< 0.001**	0.456 ± 0.469	-0.056 ± 0.533	0.2 ± 0.563
		**Trunk rotation (R_{CIRC})**	**0.016**	0.319 ± 0.488	0.518 ± 0.428	0.419 ± 0.468
		**Knee varus-valgus (R_{CIRC})**	0.344	0.49 ± 0.56	0.526 ± 0.577	0.508 ± 0.567
		**Knee rotation (R_{CIRC})**	0.717	0.167 ± 0.681	0.183 ± 0.669	0.175 ± 0.672
**Kinetic**	**Joint moments**	**Hip (R_{CIRC})**	0.371	0.661 ± 0.322	0.72 ± 0.27	0.69 ± 0.298
		**Knee (R_{CIRC})**	0.885	0.34 ± 0.254	0.34 ± 0.287	0.34 ± 0.27
		**Ankle (R_{CIRC})**	0.376	0.937 ± 0.065	0.945 ± 0.059	0.941 ± 0.062
	**Joint powers**	**Hip (R_{CIRC})**	0.266	0.494 ± 0.32	0.562 ± 0.272	0.528 ± 0.298
		**Knee (R_{CIRC})**	0.956	0.251 ± 0.427	0.245 ± 0.437	0.248 ± 0.43
		**Ankle (R_{CIRC})**	0.714	0.522 ± 0.37	0.559 ± 0.548	0.54 ± 0.346
	**Ground reaction forces**	**Anterior-posterior (R_{CIRC})**	0.865	0.836 ± 0.239	0.847 ± 0.2	0.842 ± 0.219
		**Medio-lateral (R_{CIRC})**	0.228	0.918 ± 0.045	0.931 ± 0.028	0.925 ± 0.038
		**Vertical (R_{CIRC})**	0.609	0.97 ± 0.022	0.974 ± 0.013	0.972 ± 0.018
**EMG**	**Agonist-antagonist co-activation**	**TA-GL (CoAct)**	0.592	109.585 ± 22.533	110.981 ± 28.679	110.283 ± 25.606
		**RF-ST (CoAct)**	0.903	88.353 ± 27.937	86.996 ± 28.562	87.675 ± 28.249

Legend: LS (left side), RS (right side), LS & RS (mean of left-right sides), deg (degree), SD (standard deviation), deg (degree), R_{CIRC}, (shape symmetry index), TA-GL (Tibialis anterior-gastrocnemius lateralis), RF-ST (Rectus femoris-semitendinosus), CoAct (co-activation index). Significant p-values are reported in bold.
